# Supplementary material for: USP44 regulates irradiation-induced DNA double-strand break repair and suppresses tumorigenesis in nasopharyngeal carcinoma
Source: Nat Commun. 2022 Jan 25;13:501. doi: 10.1038/s41467-022-28158-2 (PMC8789930; doi:10.1038/s41467-022-28158-2)
Supplement: Supplementary file 1 — Supplementary Information [file 41467_2022_28158_MOESM1_ESM.pdf]

Supplementary Information

USP44 regulates irradiation-induced DNA double-strand break repair and suppresses tumorigenesis in nasopharyngeal carcinoma

Chen et al.,

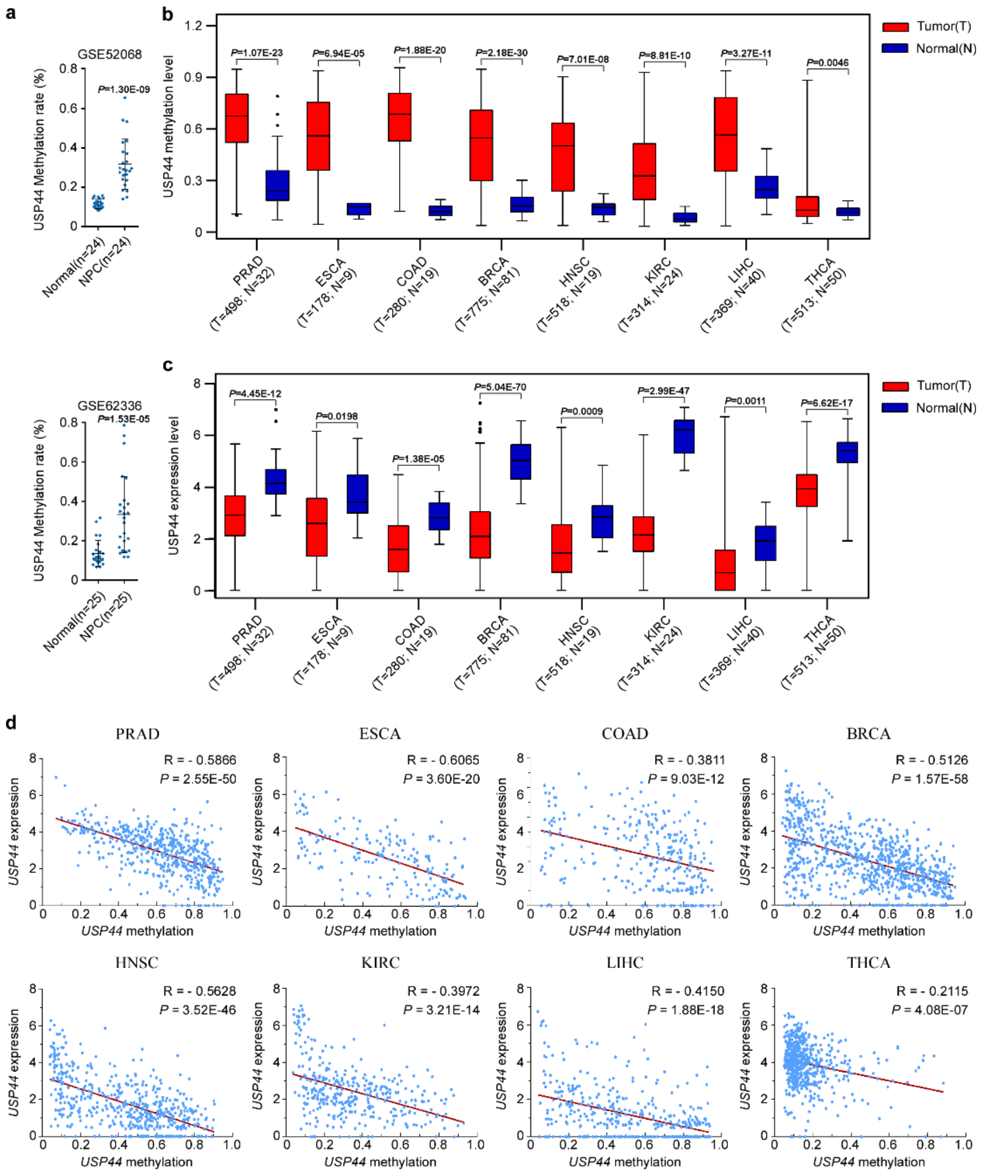

Supplementary Fig. 1 (Extended data for Main Fig. 1)

a, Methylation level of the CpG site (cg00927554) in the promoter region of *USP44* based on genome-wide methylation microarray data (GSE52068 and GSE62336). **b,c,** *USP44* methylation levels (**b**) and expression levels (**c**) in different tumors and normal tissues based on The Cancer Genome Atlas (TCGA) database are shown in box plots, with the horizontal lines representing the median; the bottom and top of the boxes representing the 25th and 75th confidence limits; and the vertical bars representing the range of data. **d,** The correlation analysis of *USP44* methylation and mRNA expression levels in different tumors from TCGA database. Data are analyzed using the spearman correlation. Abbreviations: PRAD, prostate adenocarcinoma; ESCA, esophageal carcinoma; COAD, colon adenocarcinoma; BRCA, breast invasive carcinoma; HNSC, head and neck squamous cell carcinoma; KIRC, kidney renal clear cell carcinoma; LIHC, liver hepatocellular carcinoma; THCA, thyroid carcinoma. Data in a-c as mean ± SD; the *P* values were determined using the two-tailed Student's *t*-test. Source data are provided as a Source Data file.

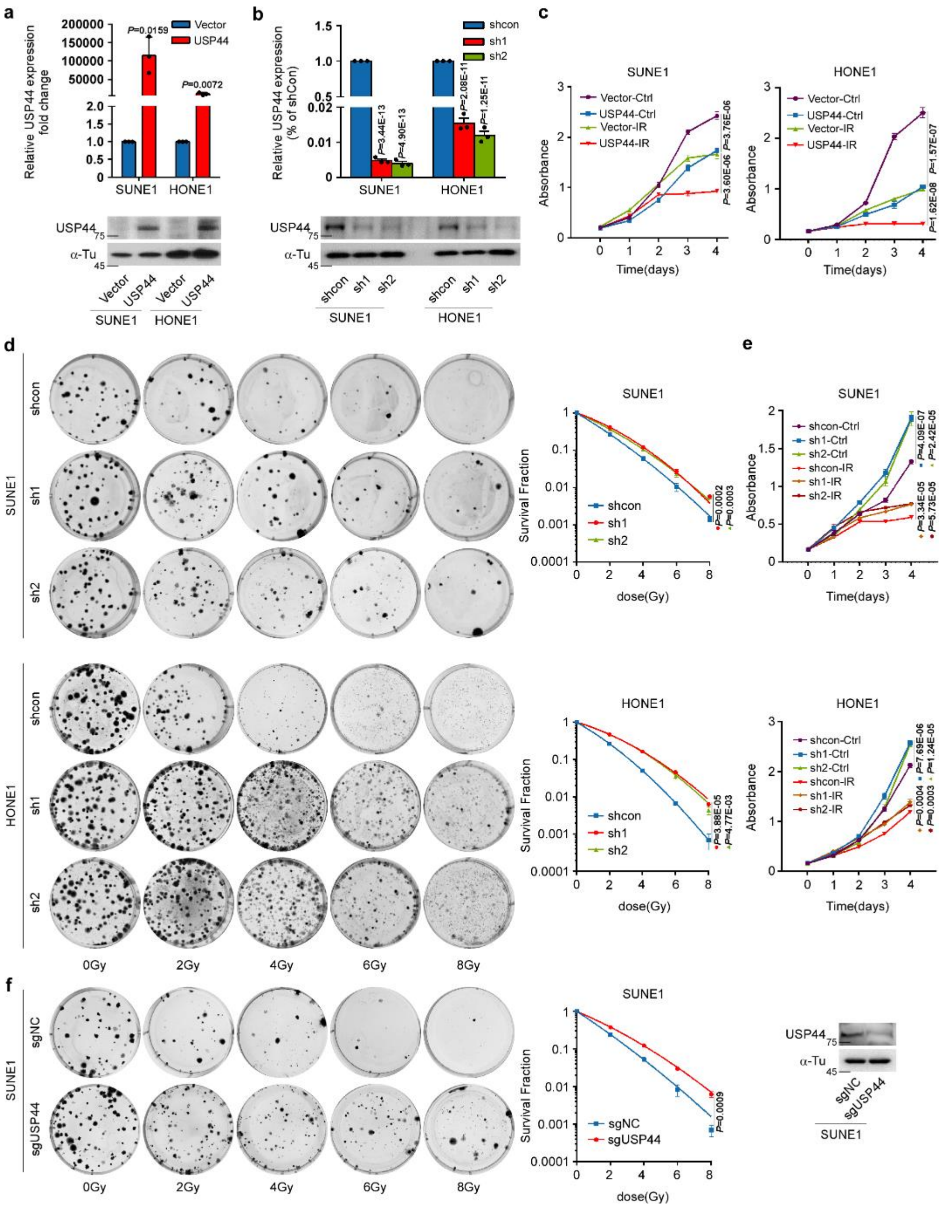

Supplementary Fig. 2 (Extended data for Main Fig. 2)

a,b, RT-PCR and western blot analysis of SUNE1 and HONE1 cells with USP44 overexpression (**a**) or knockdown (**b**). The empty vector or HA-*USP44* plasmids were stably transfected into SUNE1 or HONE1 cells. The control or sh*USP44* plasmids (sh1 or sh2) were transiently transfected into SUNE1 or HONE1 cells with stable USP44 overexpression. **c,** CCK-8 assay of SUNE1 and HONE1 cells stably transfected with *USP44* or the empty vector plasmids after exposure to 6Gy IR. **d,e,** Clonogenic assays and survival fraction curves (**d**) after exposure to indicated IR, as well as CCK-8 assay (**e**) after exposure to 6Gy IR of SUNE1 and HONE1 cells with USP44 knockdown or not. The control or sh*USP44* plasmids (sh1 or sh2) were transiently transfected into SUNE1 or HONE1 cells with stable USP44 overexpression. **f,** Clonogenic assays and survival fraction curves after exposure to indicated IR of sgNC or sg*USP44* SUNE1 cells. The sgNC or sg*USP44* cells were constructed upon SUNE1 cells with stable USP44 overexpression. Data are presented as the mean ± SD; the *P* values were determined using the two-tailed Student's *t*-test; *n* = 3 (a,b,d,f) and *n* = 4 (c,e) independent experiments. Source data are provided as a Source Data file.

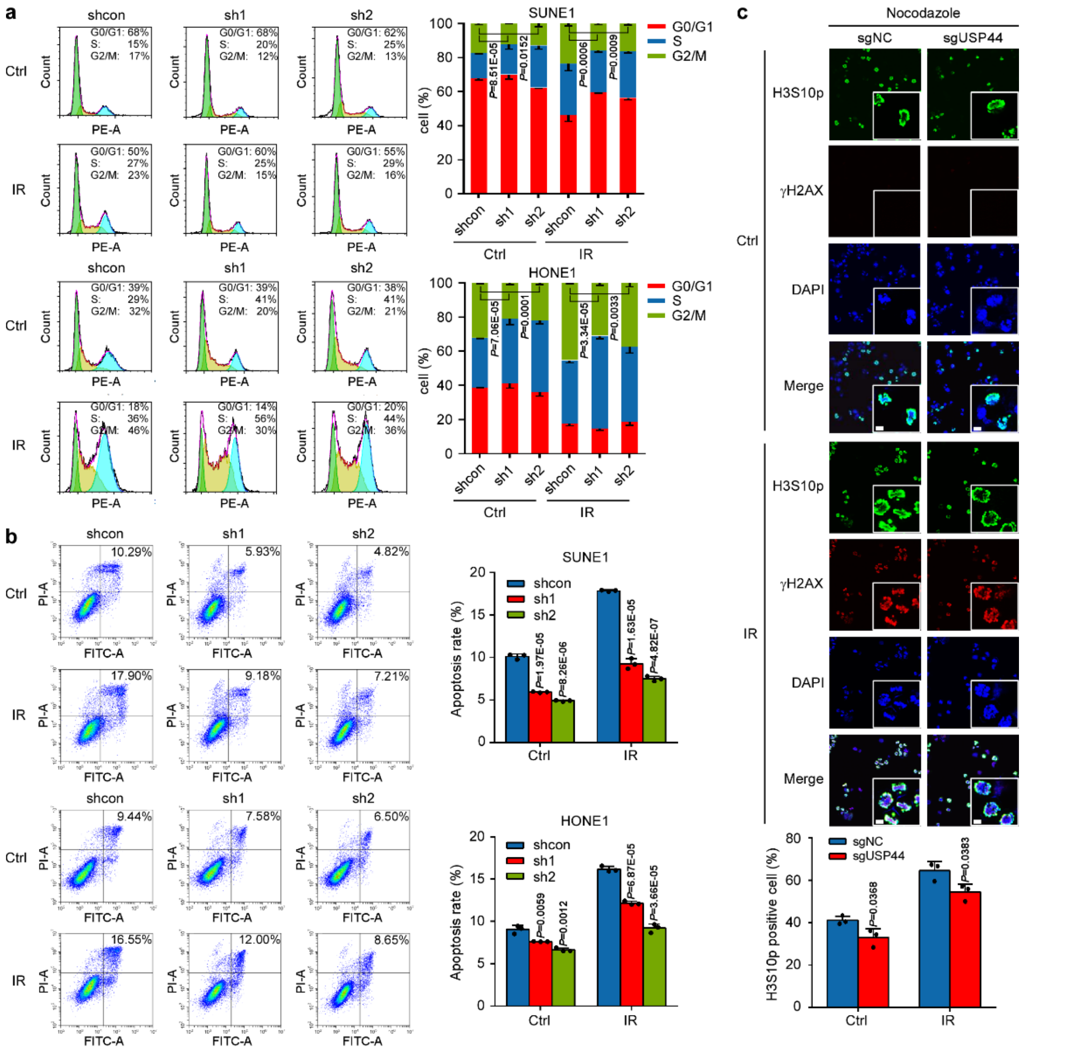

Supplementary Fig. 3 (Extended data for Main Fig. 2)

a,b, Cell cycle distribution (**a**) and apoptosis rate (**b**) of *USP44* knocked-down SUNE1 and HONE1 cells with or without exposure to 6Gy IR. The control or sh*USP44* plasmids (sh1 or sh2) were transiently transfected into SUNE1 or HONE1 cells with stable USP44 overexpression. Cell cycle distribution was detected at 8h after IR and apoptosis rate was detected at 24h after IR. **c,** The representative images and the percentage of H3S10$_p$ positive cells were analyzed by immunofluorescence staining in sgNC or sg*USP44* SUNE1 cells treated with nocodazole (0.1 μg/ml) for 18 hours before IR treatment (6 Gy, 0.5 h) or not. Scale bar, 10μm. The sgNC or sg*USP44* cells were constructed upon SUNE1 cells with stable USP44 overexpression. Data in a-c are presented as the mean ± SD; the *P* values were determined using the two-tailed Student's *t*-test; *n* = 3 independent experiments. Source data are provided as a Source Data file.

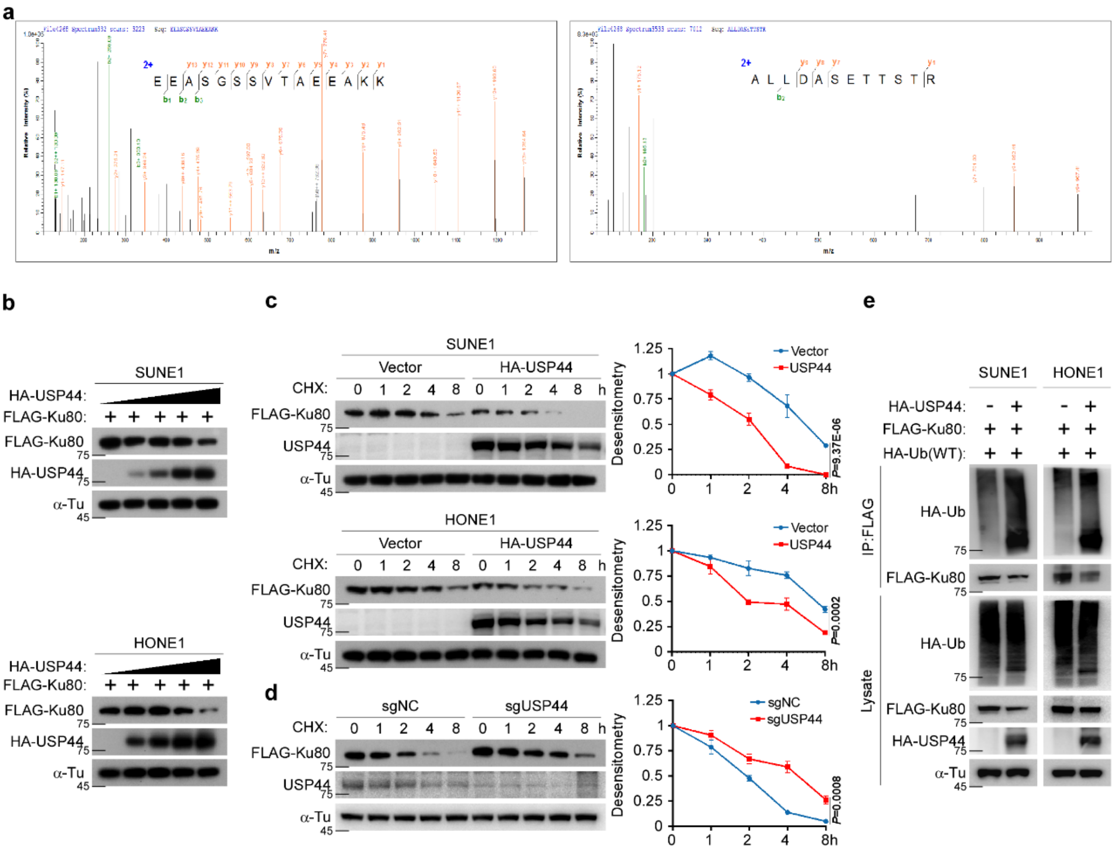

Supplementary Fig. 4 (Extended data for main Fig. 3)

a, USP44 junction-specific peptides of Ku80 (left) or TRIM25 (right) were identified with immunoprecipitation of anti-HA antibody in SUNE1 cells stably overexpressing HA-*USP44*. **b,** USP44, in a dose-dependent manner, inhibited Ku80 protein expression in SUNE1 and HONE1 cells. **c,** The effect of CHX treatment and its gray analysis in SUNE1 and HONE1 cells transfected with FLAG-*Ku80* and HA-*USP44* or the empty vector plasmids. **d,** The effect of CHX treatment and greyscale analysis of the results in sgNC or sg*USP44* SUNE1 cells transfected with FLAG-*Ku80*. **e,** SUNE1 and HONE1 cells transfected with FLAG-*Ku80*, HA-*Ub* and HA-*USP44* or the empty vector plasmids were subjected to denature-IP and immunoblotted with the indicated antibodies. Data in c are presented as the mean ± SD; the *P* values were determined using the two-tailed Student's *t*-test; *n* = 3 independent experiments. Source data are provided as a Source Data file.

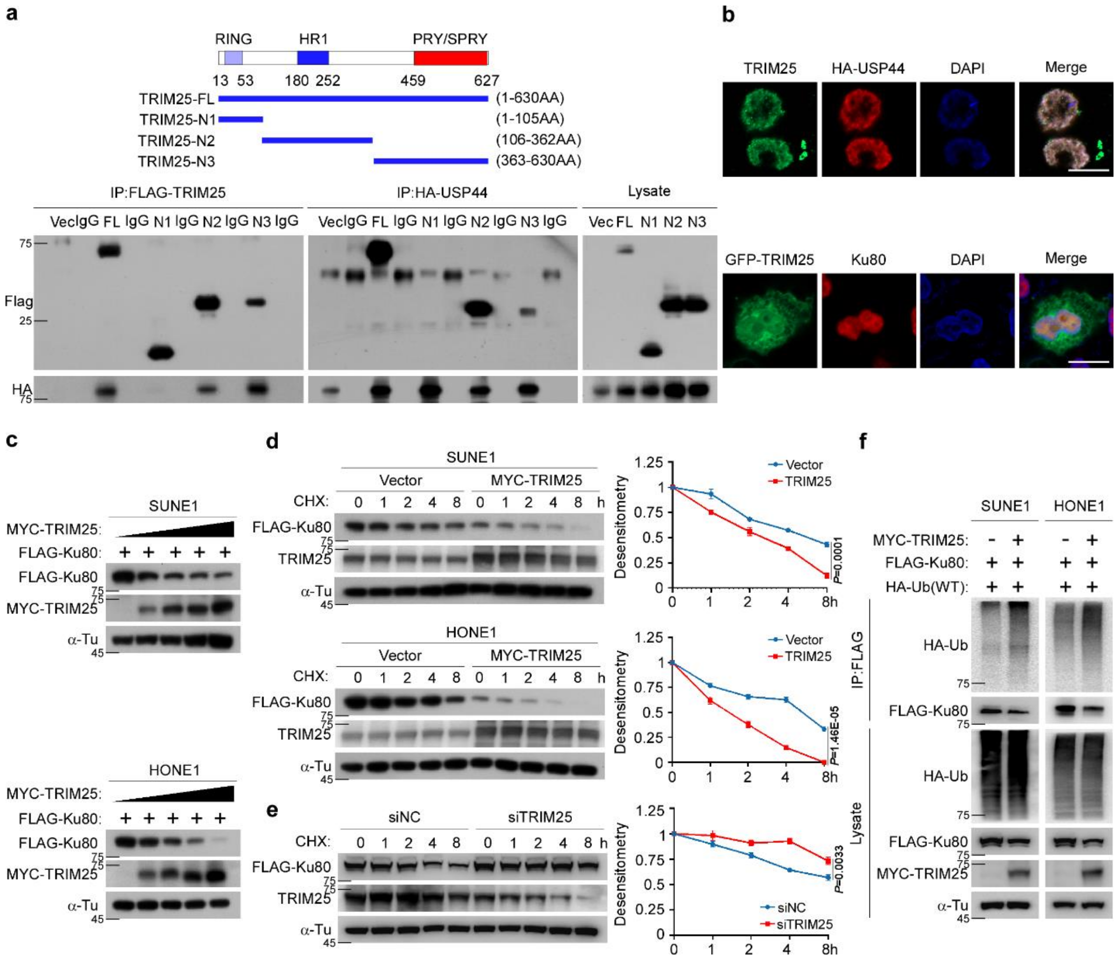

Supplementary Fig. 5 (Extended data for main Fig. 3**)**

a, A structural diagram of TRIM25 and schematic representation of Flag-tagged truncation mutants of *TRIM25*. 293T cells were transfected with HA-tagged *USP44* and Flag-tagged *TRIM25* (FL) or *TRIM25* truncation mutants. The cell lysates were collected and subjected to immunoprecipitation and immunoblotted with the indicated antibodies. **b,** Immunofluorescence staining revealed the cellular location of TRIM25 and USP44 or Ku80 in SUNE1 cells at 0.5h after exposure to 6Gy IR. Scale bars, 10 µm. **c,** TRIM25, in a dose-dependent manner, inhibited Ku80 protein expression in SUNE1 and HONE1 cells. **d,** The effect of CHX treatment and its gray analysis in SUNE1 and HONE1 cells transfected with FLAG-*Ku80* and MYC-*TRIM25* or the empty vector plasmids. **e,** The effect of CHX treatment (left) and greyscale analysis of the results (right) in HONE1 cells transfected with FLAG-*Ku80* plus siNC or si*TRIM25*. **f,** SUNE1 and HONE1 cells transfected with FLAG-*Ku80*, HA-*Ub* and MYC-*TRIM25* or the empty vector plasmids were subjected to denature-IP and immunoblotted with the indicated antibodies. Data in d-e are presented as the mean ± SD; two-tailed Student's *t*-test was used for statistical analysis; *n* = 3 independent experiments. Source data are provided as a Source Data file.

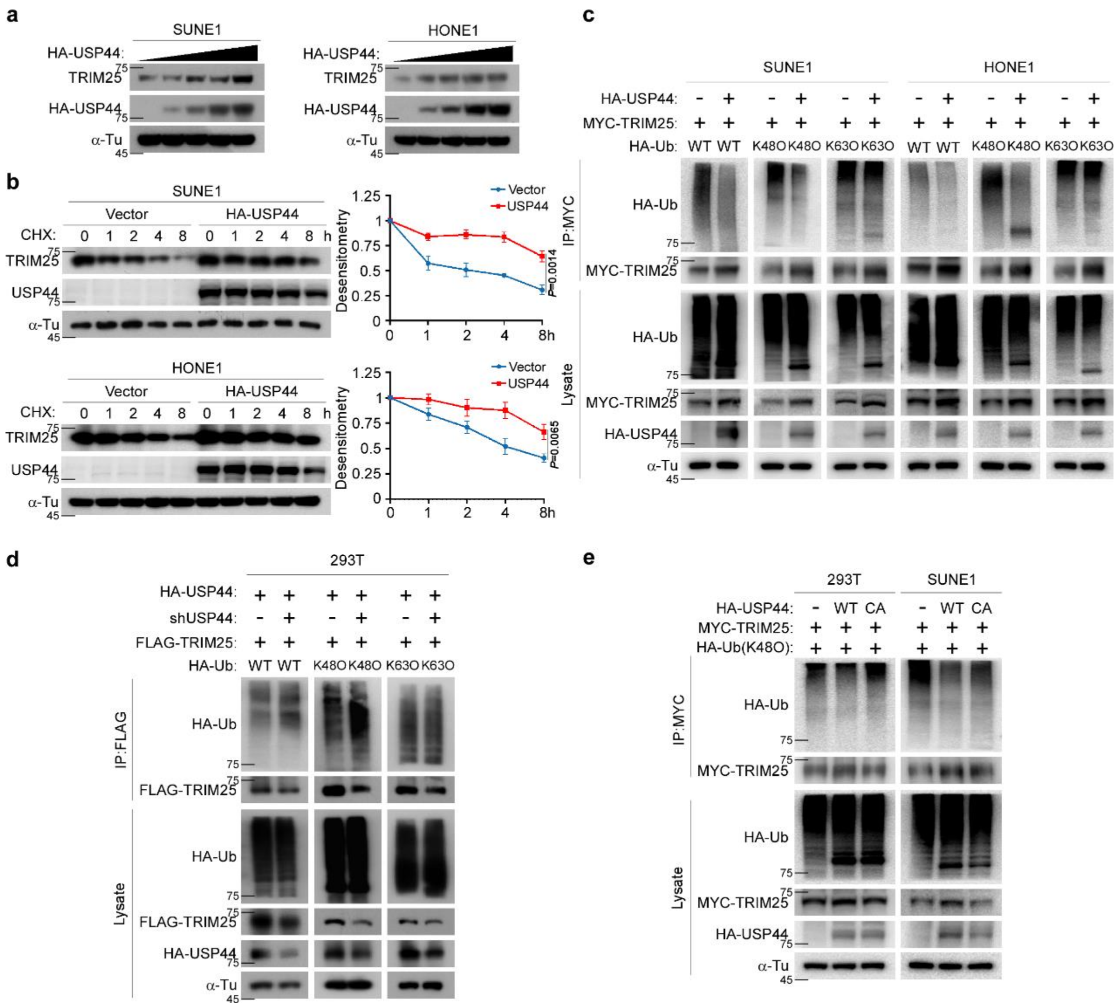

Supplementary Fig. 6 (Extended data for main Fig. 4**)**

a, USP44, in a dose-dependent manner, promoted TRIM25 protein expression in SUNE1 and HONE1 cells. **b,** The effect of CHX treatment and its gray analysis in SUNE1 and HONE1 cells transfected with HA-*USP44* or the empty vector plasmids. **c,** SUNE1 and HONE1 cells transfected with HA-*USP44* or the empty vector were co-transfected with MYC-*TRIM25* and a vector encoding HA-WT-Ub, HA-K48O-Ub or HA-K63O-Ub plasmids, were subjected to denature-IP and immunoblotted with the indicated antibodies. **d,** 293T cells equally transfected with HA-*USP44* were co-transfected with shNC or sh*USP44*, FLAG-*TRIM25* and a vector encoding HA-WT-Ub, HA-K48O-Ub or HA-K63O-Ub plasmids subjected to denature-IP and immunoblotted with the indicated antibodies. **e,** 293T and SUNE1 cells transfected with the vector plasmid, HA-*USP44* or HA-*USP44*(C282A) together with MYC-*TRIM25* and HA-K48O-Ub were subjected to denature-IP and immunoblotted with the indicated antibodies. Data in b are presented as the mean ± SD; the *P* values were determined using the two-tailed Student's *t*-test; *n* = 3 independent experiments. Source data are provided as a Source Data file.

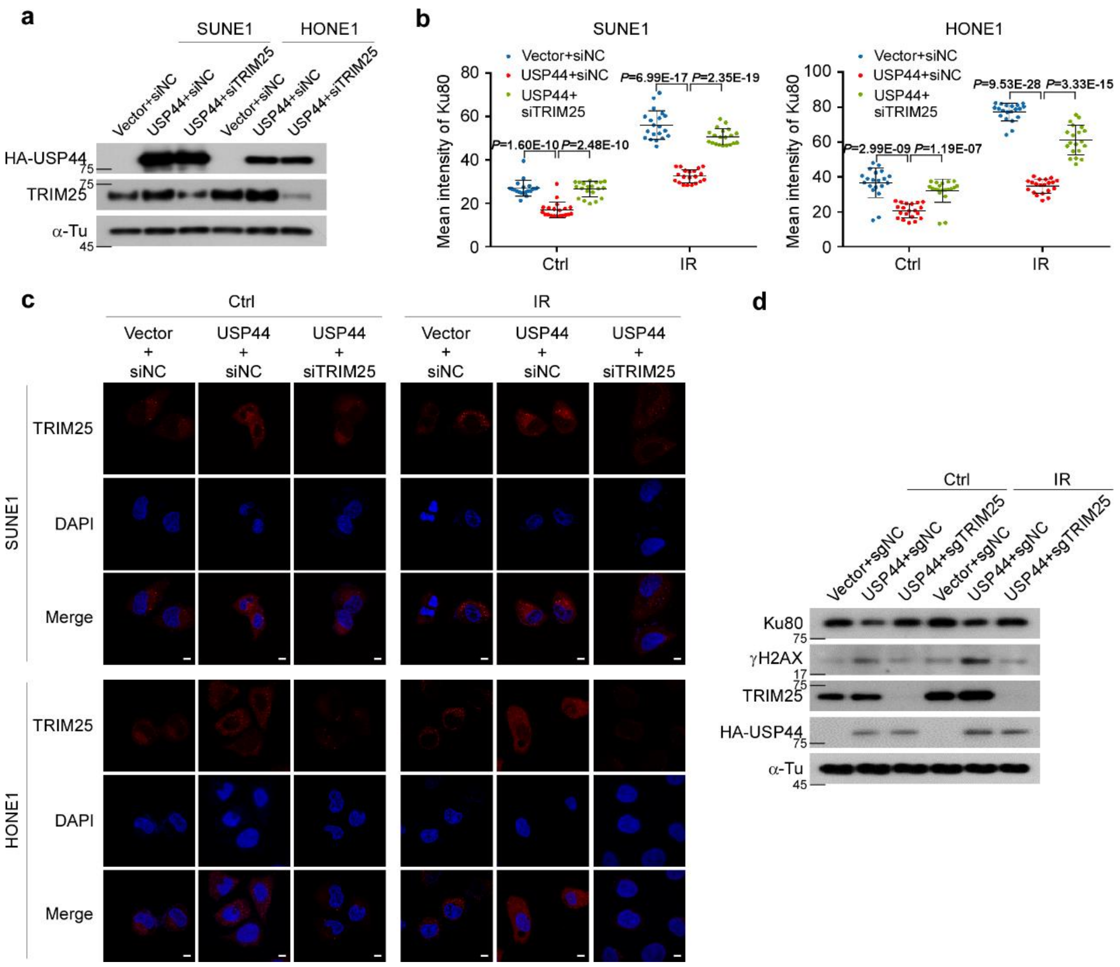

Supplementary Fig. 7 (Extended data for Main Fig. 4)

a, The expression efficiency of NPC cells transfected with the indicated plasmids and siRNAs detected by the western blotting assay. **b,** The mean intensity of Ku80 fluorescence (n = 20 cells per group) in Fig. 4j was quantified using ImageJ software. **c,** SUNE1 and HONE1 cells exposed to IR (6 Gy) transfected with the indicated plasmids and siRNAs were fixed 0.5 h later and co-immunostained with the TRIM25 antibody. Scale bars, 10 μm. **d,** Western blot analysis of the Vector+sgNC, *USP44*+sgNC, and *USP44*+sg*TRIM25* grouped SUNE1 cells with IR treatment (6 Gy, 0.5 h) or not. Data in b are presented as the mean ± SD; the P values were determined using the two-tailed Student's t-test; n = 3 independent experiments. Source data are provided as a Source Data file.

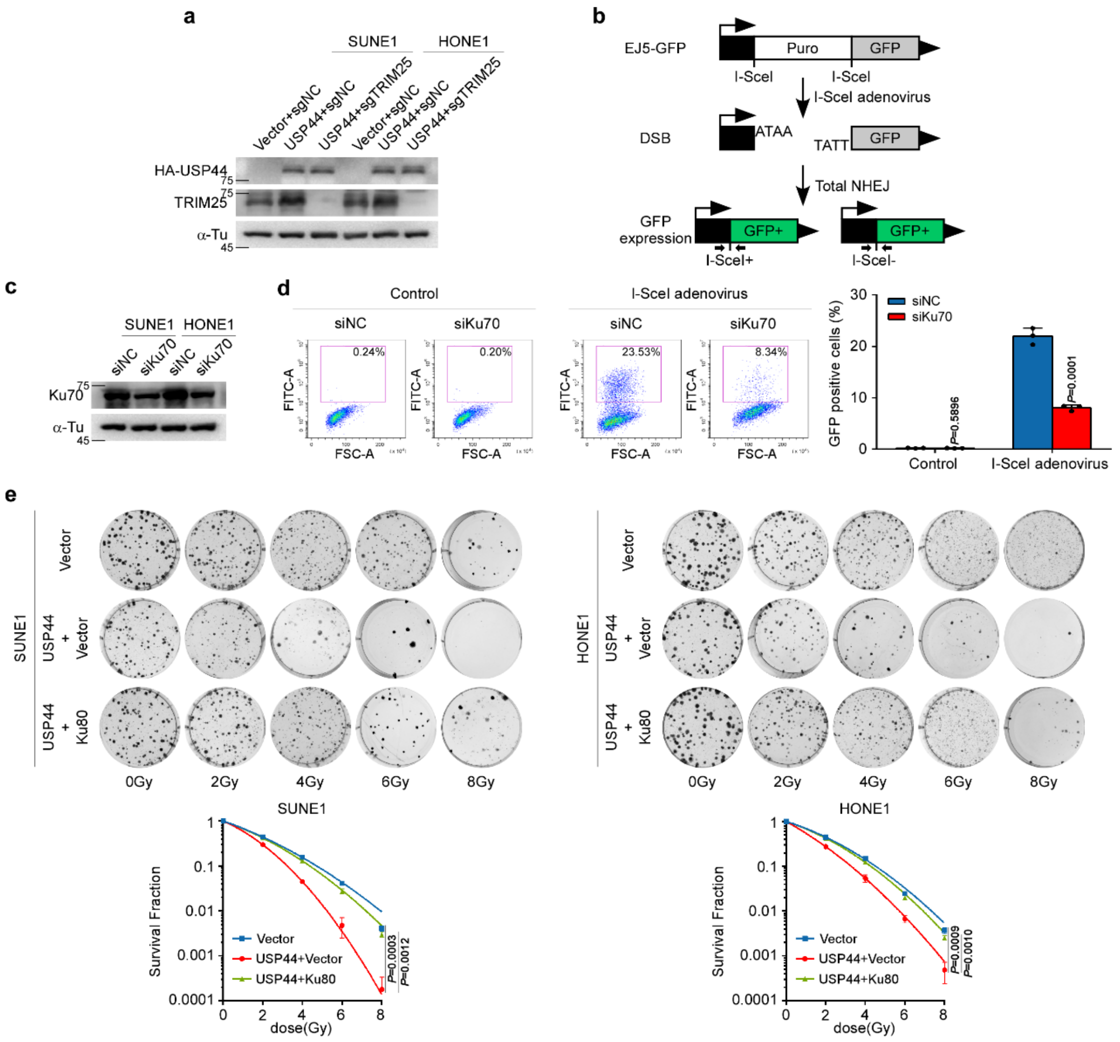

Supplementary Fig. 8 (Extended data for Main Fig. 5-6)

a, Western blot analysis of the Vector+sgNC, *USP44*+sgNC, and *USP44*+sg*TRIM25* grouped SUNE1 and HONE1 cells. **b,** Schematic of the EJ5-GFP reporter used to monitor NHEJ repair in NPC cells (see text for details). **c,** Western blot analysis of SUNE1 and HONE1 cells transfected with siNC or si*Ku70*. **d,** The siNC or si*Ku70* SUNE1 cells were transfected with EJ5-GFP, infected with or without I-SceI-expressing adenovirus and analysed for GFP positivity by flow cytometry. **e,** Clonogenic assays and survival fraction curves after exposure to indicated IR of SUNE1 and HONE1 cells transiently co-transfected with HA-*USP44* or the empty vector plus FLAG-*TRIM25* or the empty vector plasmids. Data in d,e are presented as the mean ± SD; the P values were determined using the two-tailed Student's t-test; n = 3 independent experiments. Source data are provided as a Source Data file.

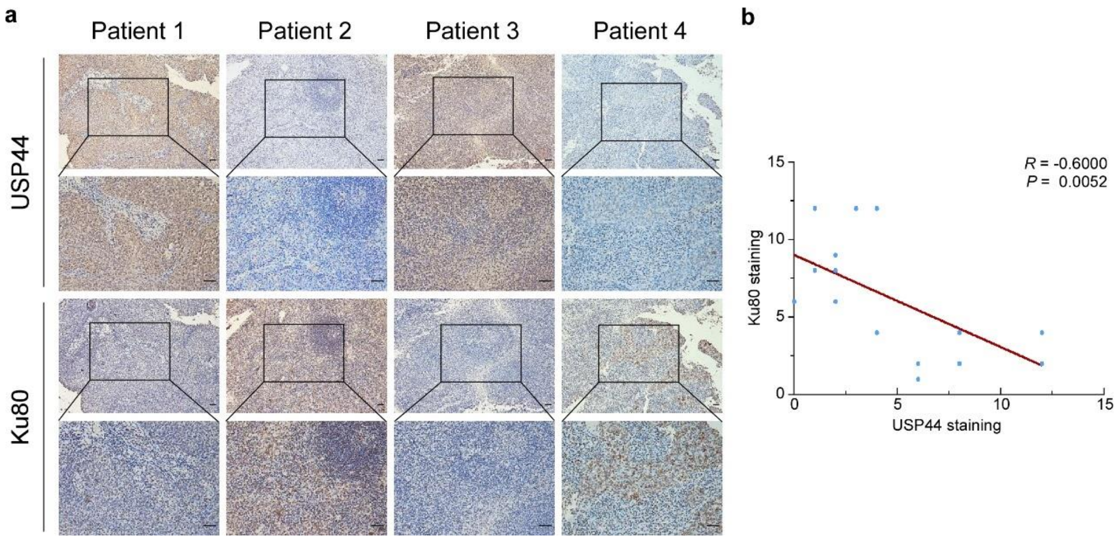

Supplementary Fig. 9 (Extended data for Main Fig. 8**)**

a, Representative images of immunohistochemical staining anti-USP44 or anti-Ku80 in 20 NPC patients. Scale bars, 50 μm. **b,** Correlation analysis of USP44 expression and Ku80 expression in NPC samples (*n* = 20) according to IHC score statistics. Data are analyzed using the spearman correlation. Source data are provided as a Source Data file.

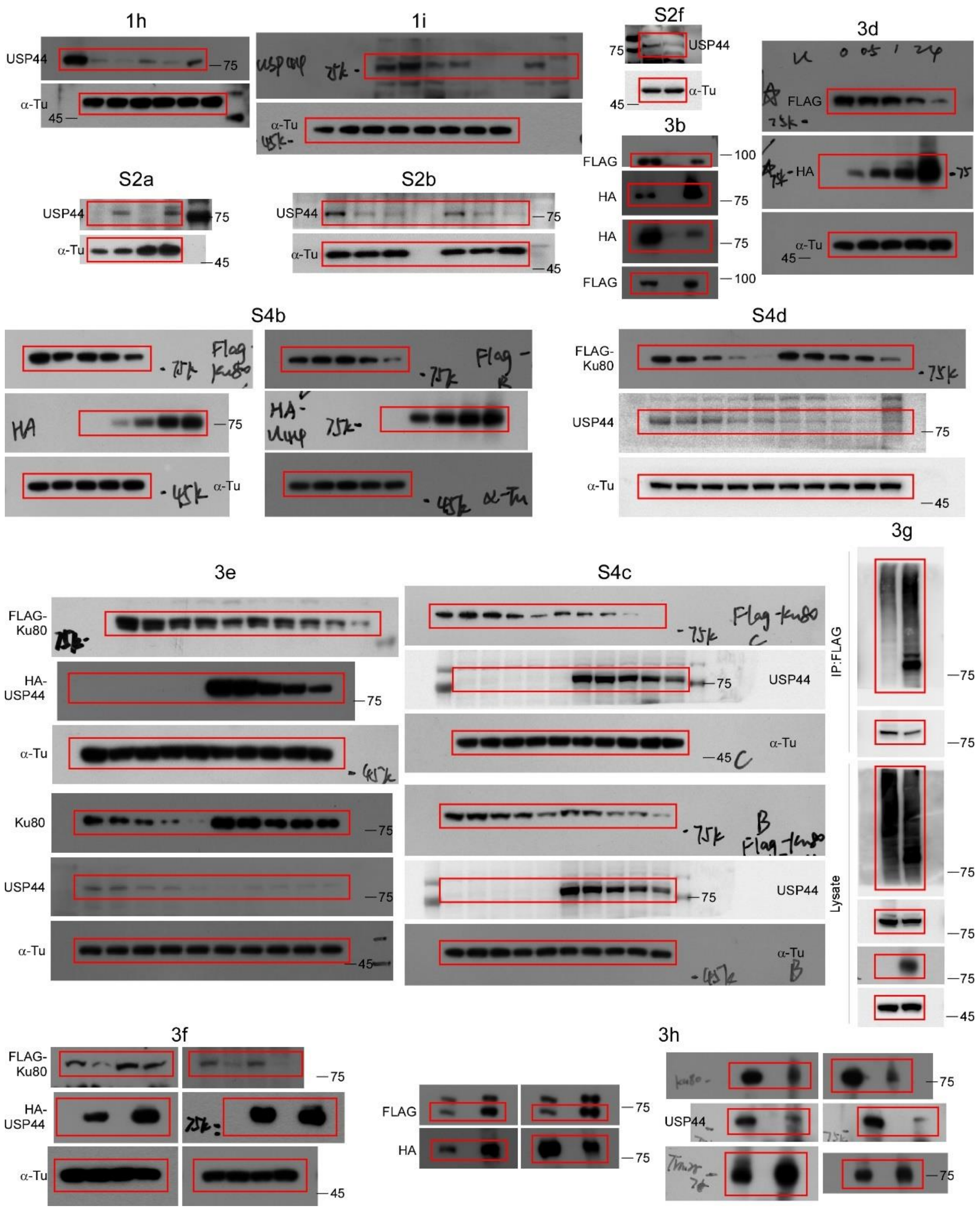

Supplementary Fig. 10 (Unprocessed immunoblots)

Unprocessed immunoblots for indicated Figures panels.

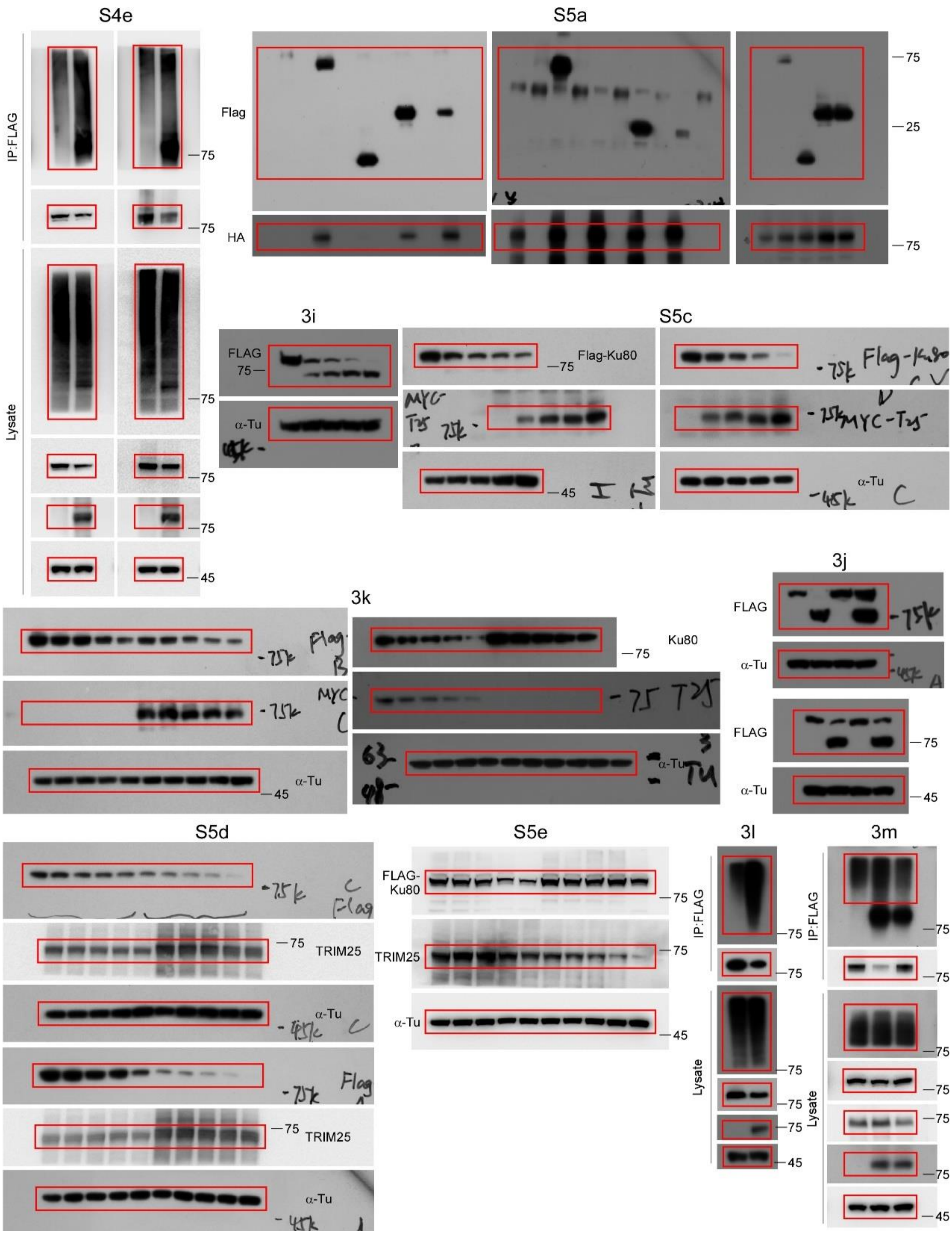

Supplementary Fig. 10 (cont'd) (Unprocessed immunoblots (continued)**)**
Unprocessed immunoblots for indicated Figures panels.

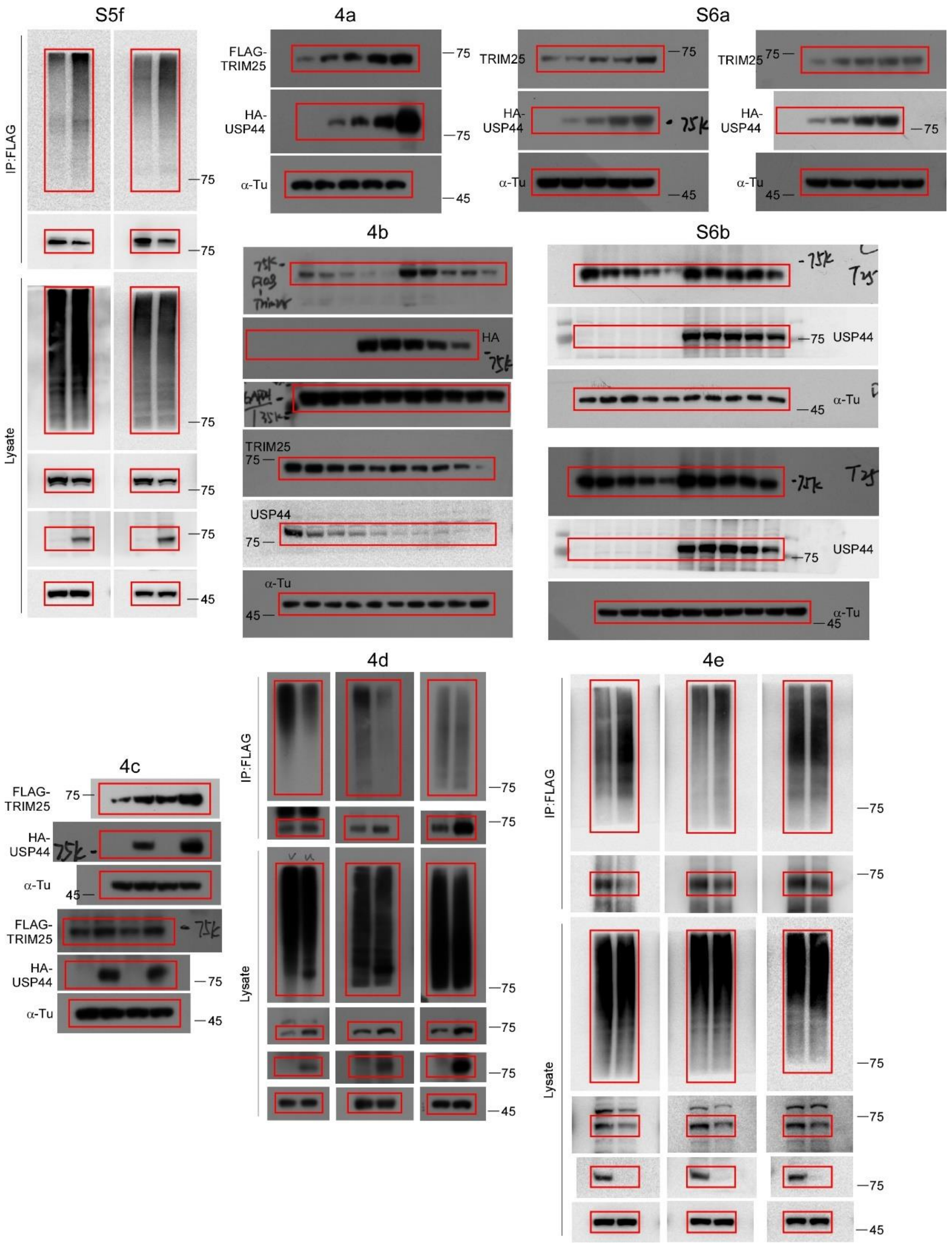

Supplementary Fig. 10 (cont'd) (Unprocessed immunoblots (continued)**)**
Unprocessed immunoblots for indicated Figures panels.

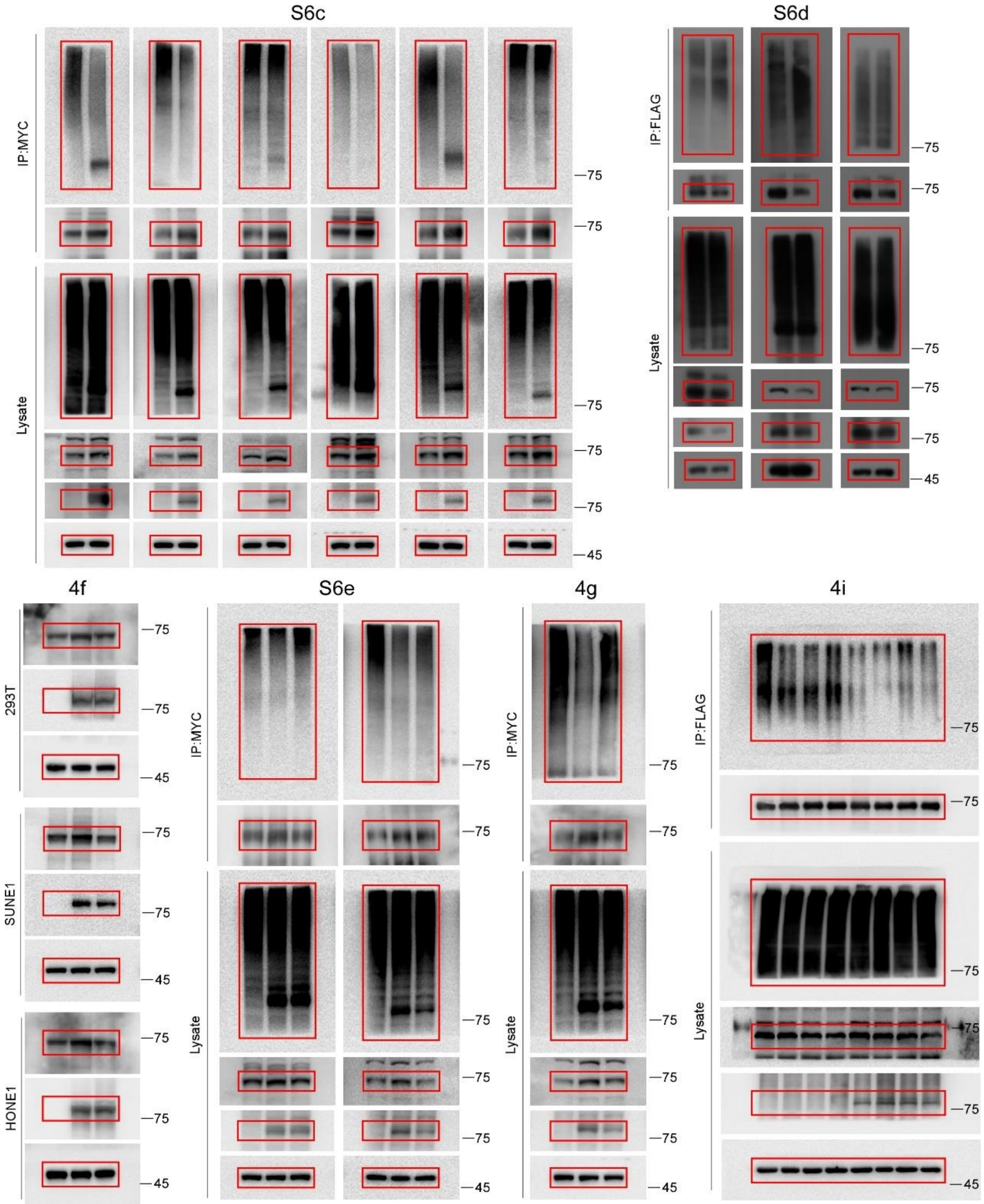

Supplementary Fig. 10 (cont'd) (Unprocessed immunoblots (continued)**)**
Unprocessed immunoblots for indicated Figures panels.

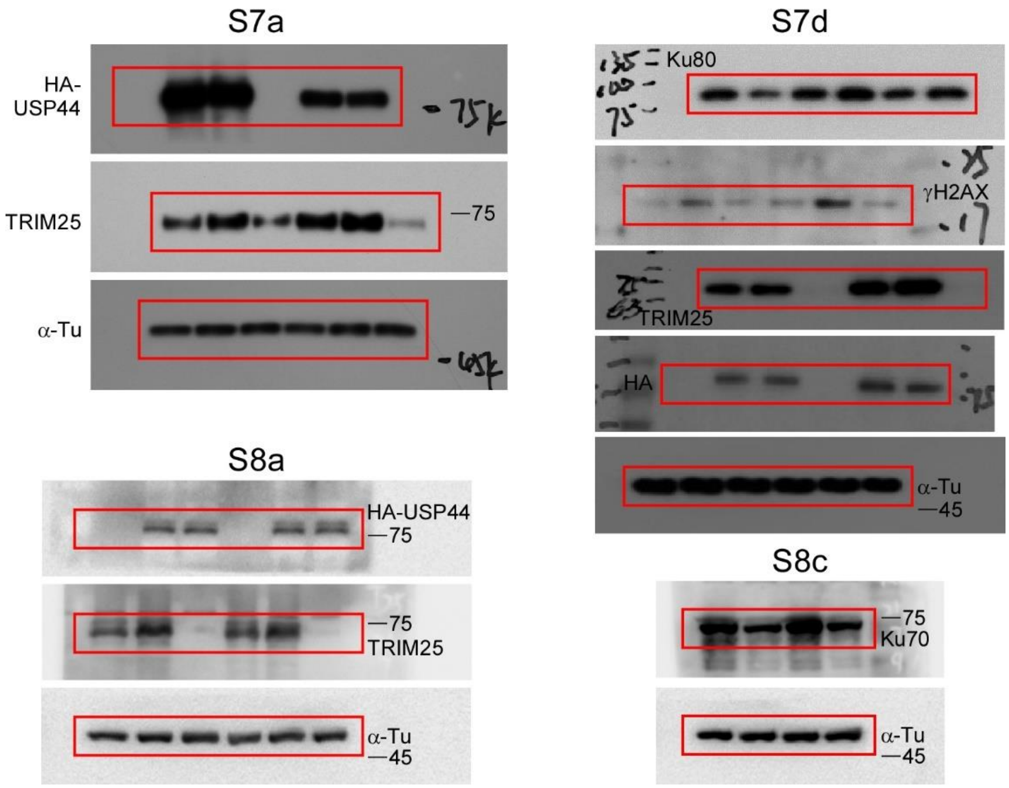

Supplementary Fig. 10 (cont'd) (Unprocessed immunoblots (continued)**)**
Unprocessed immunoblots for indicated Figures panels.

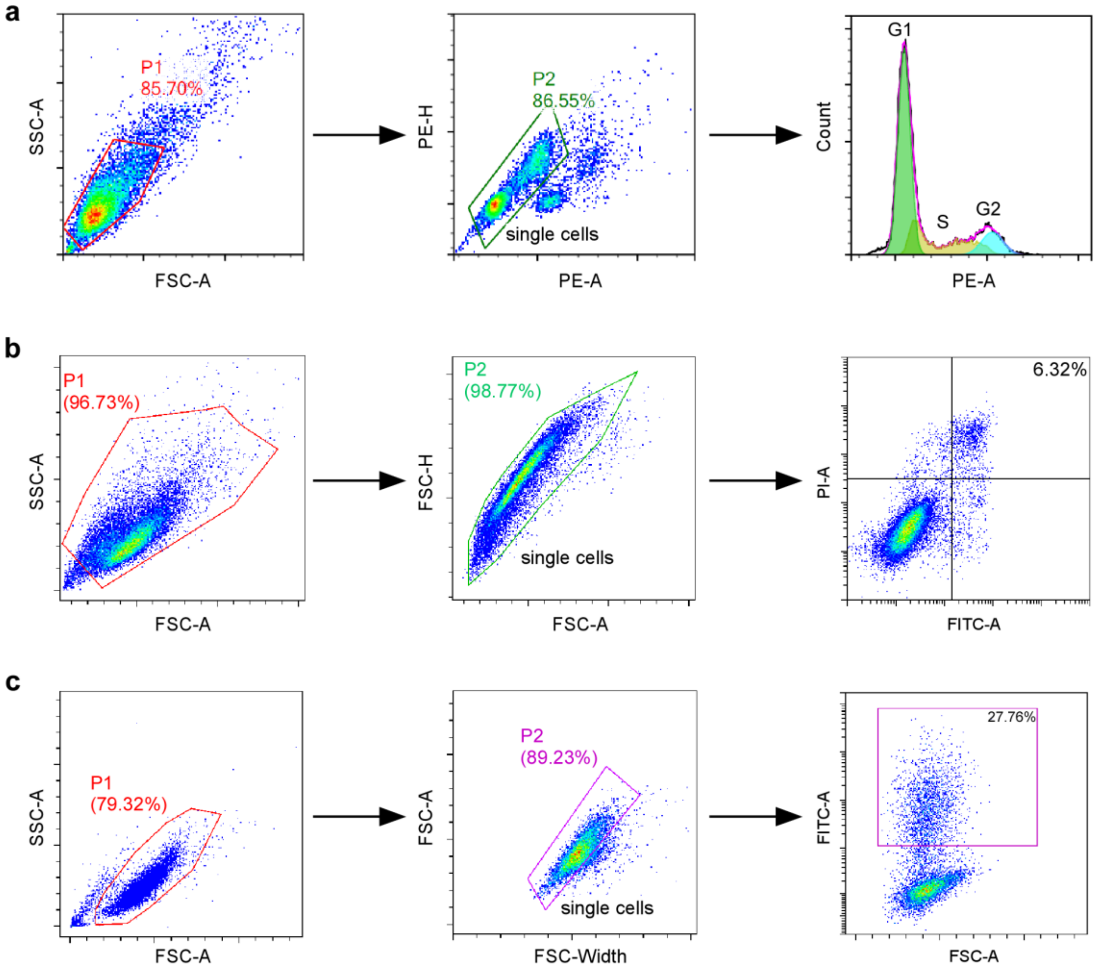

Supplementary Fig. 11

The gating strategy of Flow cytometry analysis

Supplementary Table 1. The USP44-interacting proteins identified by mass spectrometry in SUNE1 cells with stable USP44 overexpression.

Accession	Protein description	Score	Matches
A0A024RBD7_HUMAN	Ubiquitin hydrolase 44	3259	165
A0A024R1N4_HUMAN	X-ray repair complementing defective repair in Chinese hamster cells 6	2709	114
XRCC5_HUMAN	X-ray repair cross-complementing protein 5	2036	125
HSP7C_HUMAN	Heat shock cognate 71	1921	97
LMNA_HUMAN	Prelamin-A/C	1392	70
A0A1U9X7W4_HUMAN	HSPA1A	1116	58
A0A024R9C1_HUMAN	Polyadenylate-binding protein	668	32
HNRPM_HUMAN	Heterogeneous nuclear ribonucleoprotein M	614	43
BIP_HUMAN	Endoplasmic reticulum chaperone BiP	597	27
LAP2A_HUMAN	Lamina-associated polypeptide 2, isoform alpha	597	24
B2R6X5_HUMAN	cDNA, FLJ93166, highly similar to Homo sapiens heat shock 70kDa protein 6	548	27
HNRPU_HUMAN	Heterogeneous nuclear ribonucleoprotein U	480	21
B7Z4V2_HUMAN	cDNA FLJ51907, highly similar to Stress-70 protein, mitochondrial	446	18
A0A0D9SF53_HUMAN	ATP-dependent RNA helicase DDX3X	442	30
DHX9_HUMAN	ATP-dependent RNA helicase A	388	29
H3BNC9_HUMAN	Uncharacterized protein	386	21
A0A024RCT9_HUMAN	High mobility group AT-hook 1, isoform CRA_b	351	19
A0A024R228_HUMAN	Heterogeneous nuclear ribonucleoprotein K, isoform CRA_d	328	16
B4DN41_HUMAN	cDNA FLJ53366, highly similar to Probable ATP-dependent RNA helicase DDX5	300	18
A0A0S2Z428_HUMAN	HCG2039812, isoform CRA_b (Fragment)	299	19
A0A1W2PQ51_HUMAN	Probable ATP-dependent RNA helicase DDX17	295	17
A0A0C4DGB6_HUMAN	Serum albumin	265	16
RS18_HUMAN	40S ribosomal protein S18	231	8
A0A024RD80_HUMAN	Heat shock protein 90kDa alpha (Cytosolic), class B member 1, isoform CRA_a	212	14
D7RXV9_HUMAN	DING protein p38	202	4
A0A024R609_HUMAN	Pyruvate kinase	198	10
A0A024R5Z9_HUMAN	Pyruvate kinase	197	11
EZRI_HUMAN	Ezrin	197	11
A0A0S2Z4Z0_HUMAN	RNA binding motif protein 14 isoform 1 (Fragment)	184	10
RBP56_HUMAN	TATA-binding protein-associated factor 2N	150	4

Accession	Protein description	Score	Matches
A0A024R4F1_HUMAN	Enolase 1, (Alpha), isoform CRA_a	142	9
RPN1_HUMAN	Dolichyl-diphosphooligosaccharide--protein glycosyltransferase subunit 1	140	9
RL12_HUMAN	60S ribosomal protein L12	140	6
MOES_HUMAN	Moesin OS=Homo sapiens	139	7
HS90A_HUMAN	Heat shock protein HSP 90-alpha	138	9
A0A075B6Z2_HUMAN	T cell receptor alpha joining 56 (Fragment)	137	26
RS25_HUMAN	40S ribosomal protein S25 OS=Homo sapiens	136	8
F8WJN3_HUMAN	Cleavage and polyadenylation-specificity factor subunit 6	136	4
E9PK25_HUMAN	Cofilin-1	129	4
RL35_HUMAN	60S ribosomal protein L35	128	7
K7EJ78_HUMAN	40S ribosomal protein S15	120	8
ACTB_HUMAN	Actin, cytoplasmic 1	117	5
MISP_HUMAN	Mitotic interactor and substrate of PLK1	104	6
RS13_HUMAN	40S ribosomal protein S13	103	7
TCPG_HUMAN	T-complex protein 1 subunit gamma	102	5
B1ANR0_HUMAN	Polyadenylate-binding protein	99	5
K7EJV9_HUMAN	60S ribosomal protein L23a (Fragment)	98	2
GRN_HUMAN	Granulins	96	5
V9HW68_HUMAN	Epididymis luminal protein 214	90	12
A0A024R3X4_HUMAN	Heat shock 60kDa protein 1 (Chaperonin), isoform CRA_a	87	3
B4E022_HUMAN	cDNA FLJ56274, highly similar to Transketolase (EC 2.2.1.1)	86	3
A0A087WVQ9_HUMAN	Elongation factor 1-alpha 1	85	3
TCPD_HUMAN	T-complex protein 1 subunit delta	84	3
M0QZC5_HUMAN	40S ribosomal protein S11	83	8
A0A024R4A0_HUMAN	Nucleolin, isoform CRA_b	81	5
A0A0F7KYT8_HUMAN	Fragile X mental retardation autosomal homolog variant p2K	79	3
LPPRC_HUMAN	Leucine-rich PPR motif-containing protein, mitochondrial	79	2
ANM5_HUMAN	Protein arginine N-methyltransferase 5	76	8
A0A024RA28_HUMAN	Heterogeneous nuclear ribonucleoprotein A2/B1, isoform CRA_d	74	1
TCPB_HUMAN	T-complex protein 1 subunit beta	72	2
A0A0G2JPR0_HUMAN	Complement C4-A	71	2
H0YKD8_HUMAN	60S ribosomal protein L28	71	3
HORN_HUMAN	Hornerin	70	3
A0A1W2PQM2_HUMAN	Tubulin alpha-1C chain	69	2
NDKA_HUMAN	Nucleoside diphosphate kinase A	68	5

Accession	Protein description	Score	Matches
Q08ES8_HUMAN	Cell growth-inhibiting protein 34	67	2
TCPH_HUMAN	T-complex protein 1 subunit eta	65	3
A0A140VJW2_HUMAN	Stathmin	63	1
A0A0S2Z4A5_HUMAN	DNA replication licensing factor MCM7 (Fragment)	63	3
B4DDU6_HUMAN	cDNA FLJ50442, highly similar to T-complex protein 1 subunit epsilon	62	3
HNRPL_HUMAN	Heterogeneous nuclear ribonucleoprotein L	61	3
PPIB_HUMAN	Peptidyl-prolyl cis-trans isomerase B	58	4
EF2_HUMAN	Elongation factor 2	58	4
HSF2_HUMAN	Heat shock factor protein 2	55	2
A0A0S2Z4Z9_HUMAN	Non-POU domain containing octamer-binding isoform 1 (Fragment)	52	2
RS10_HUMAN	40S ribosomal protein S10	52	3
Q2TSD0_HUMAN	Glyceraldehyde-3-phosphate dehydrogenase	51	1
SYTC_HUMAN	Threonine--tRNA ligase, cytoplasmic	49	1
A0A024QZX3_HUMAN	Serpin peptidase inhibitor, clade B (Ovalbumin), member 6, isoform CRA_a	48	1
A0A087WTP3_HUMAN	Far upstream element-binding protein 2	48	3
H0Y2W2_HUMAN	ATPase family AAA domain-containing protein 3A (Fragment)	47	3
Q59ET3_HUMAN	Chaperonin containing TCP1, subunit 6A isoform a variant (Fragment)	47	1
H0Y4R1_HUMAN	Inosine-5'-monophosphate dehydrogenase 2 (Fragment)	47	2
A0A024R2T6_HUMAN	DEAH (Asp-Glu-Ala-His) box polypeptide 30, isoform CRA_b	47	3
ATX2L_HUMAN	Ataxin-2-like protein	45	4
TCPQ_HUMAN	T-complex protein 1 subunit theta	44	1
E9PBF6_HUMAN	Lamin-B1	43	4
J3QRS3_HUMAN	Myosin regulatory light chain 12A	43	2
A0A2P9AP59_HUMAN	Uncharacterized protein	42	1
TCPA_HUMAN	T-complex protein 1 subunit alpha	42	3
A0A024R599_HUMAN	Solute carrier family 3 (Activators of dibasic and neutral amino acid transport), member 2, isoform CRA_e	41	2
A0A2R8Y5P9_HUMAN	Protein Shroom3	41	3
TCP4_HUMAN	Activated RNA polymerase II transcriptional coactivator p15	41	2
VIME_HUMAN	Vimentin	41	2
L0R5A1_HUMAN	Alternative protein CSF2RB	40	2

Accession	Protein description	Score	Matches
B4DP70_HUMAN	cDNA FLJ51017, highly similar to ATP-dependent RNA helicase DDX1 (EC 3.6.1.-) (Fragment)	40	2
A0A024RDB2_HUMAN	Ras-GTPase activating protein SH3 domain-binding protein 2, isoform CRA_b	40	1
A0A087X2G1_HUMAN	ATP-dependent RNA helicase DDX1	39	3
A0A024R0Y6_HUMAN	ADP-ribosylation factor 3, isoform CRA_b	39	1
B4DWL1_HUMAN	cDNA FLJ59240, highly similar to Far upstream element-binding protein 1	39	1
A0A090N8Y2_HUMAN	Protein disulfide-isomerase A4	39	1
A0A024R1N1_HUMAN	Myosin, heavy polypeptide 9, non-muscle, isoform CRA_a	38	4
NSUN2_HUMAN	tRNA (cytosine(34)-C(5))-methyltransferase	38	1
D3DTW3_HUMAN	Insulin-like growth factor 2 mRNA binding protein 1 deltaN CRDBP	37	1
J3KR24_HUMAN	Isoleucine--tRNA ligase, cytoplasmic	36	2
THIO_HUMAN	Thioredoxin	36	1
B2RU06_HUMAN	eIF4G1 protein	35	1
A0A0B4J220_HUMAN	Chromosome 11 open reading frame 48, isoform CRA_c	35	2
A0A2Q2TH77_HUMAN	Golgin subfamily A member 2	35	3
TR150_HUMAN	Thyroid hormone receptor-associated protein 3	35	1
A0A075B793_HUMAN	GRIP1-associated protein 1	34	1
A0A024R321_HUMAN	Filamin B, beta (Actin binding protein 278), isoform CRA_a	34	1
CEBPB_HUMAN	CCAAT/enhancer-binding protein beta	33	1
A0A024QZD1_HUMAN	Ribosomal protein L18, isoform CRA_c	33	1
WDR1_HUMAN	WD repeat-containing protein 1	32	1
ABCAA_HUMAN	ATP-binding cassette sub-family A member 10	32	1
A1L4G8_HUMAN	C18orf34 protein	32	1
SF3B3_HUMAN	Splicing factor 3B subunit 3	32	1
ZN326_HUMAN	DBIRD complex subunit ZNF326	32	1
SF3B2_HUMAN	Splicing factor 3B subunit 2	32	1
PLEC_HUMAN	Plectin	31	1
HNRPR_HUMAN	Heterogeneous nuclear ribonucleoprotein R	31	3
ASGR1_HUMAN	Asialoglycoprotein receptor 1	29	1
H3BRD5_HUMAN	Unconventional myosin-IXa	29	1
Q6MZK8_HUMAN	Uncharacterized protein DKFZp686K06110	28	2
H0Y403_HUMAN	Juxtaposed with another zinc finger protein 1 (Fragment)	28	2
SYRC_HUMAN	Arginine--tRNA ligase, cytoplasmic	28	2

Accession	Protein description	Score	Matches
B4DN52_HUMAN	cDNA FLJ55990, highly similar to LIM domain and actin-binding protein 1	28	1
A0A0A0MSM0_HUMAN	Heat shock protein 105 kDa	27	1
MMP3_HUMAN	Stromelysin-1	27	1
B4DUF2_HUMAN	cDNA FLJ50227, moderately similar to Complement decay-accelerating factor	27	1
Q8N9M2_HUMAN	cDNA FLJ36887 fis, clone BNGH42005504, highly similar to 26S PROTEASOME REGULATORY SUBUNIT S3	27	2
E1CKY7_HUMAN	Protein phosphatase 1 regulatory subunit 12B	27	1
D2SNZ4_HUMAN	Proline rich 11, isoform CRA_a	27	1
KHDR1_HUMAN	KH domain-containing, RNA-binding, signal transduction-associated protein 1	27	1
B7Z1Q2_HUMAN	cDNA FLJ61102, highly similar to Growth-arrest-specific protein 7	27	1
A0A2P9AHE1_HUMAN	Putative oxidoreductase with FAD-binding domain	26	1
A0A024R8I8_HUMAN	Chromosome 9 open reading frame 140, isoform CRA_a	26	1
CO6A5_HUMAN	Collagen alpha-5(VI) chain	26	1
A0A2P9AXY1_HUMAN	Excinuclease ABC subunit C (Modular protein)	25	1
RAB20_HUMAN	Ras-related protein Rab-20	24	1
A0A2P9AUT9_HUMAN	Adenine deaminase	24	1
A0A2P9ATJ5_HUMAN	Error-prone DNA polymerase 2	24	1
PYRG1_HUMAN	CTP synthase 1	24	1
A0A2P9AG47_HUMAN	Transcriptional regulator	23	1
A0A090N7X1_HUMAN	ATP-binding cassette, sub-family F (GCN20), member 2	23	1
ZN723_HUMAN	Zinc finger protein 723	22	1
TRI25_HUMAN	E3 ubiquitin/ISG15 ligase TRIM25	22	1
A0A2P9AAS4_HUMAN	Oxidoreductase	22	1
B3KPZ3_HUMAN	cDNA FLJ32490 fis, clone SKNSH1000301, highly similar to Epidermal growth factor receptor kinasesubstrate 8-like protein 1	22	1
Q59HF2_HUMAN	D site of albumin promoter (Albumin D-box) binding protein variant (Fragment)	21	1
Q8N7E8_HUMAN	cDNA FLJ25713 fis, clone TST05089	20	1
A0A2P9AIR9_HUMAN	Putative transcriptional regulator	20	1
FIP1_HUMAN	Pre-mRNA 3'-end-processing factor FIP1	18	1

Accession	Protein description	Score	Matches
H3BPW6_HUMAN	Obscurin (Fragment)	17	1
A0A087X2D0_HUMAN	Serine/arginine-rich-splicing factor 3	17	1
ATRX_HUMAN	Transcriptional regulator ATRX	17	1
H0Y8L8_HUMAN	Probable ATP-dependent RNA helicase DDX41 (Fragment)	16	1
E2AK2_HUMAN	Interferon-induced, double-stranded RNA-activated protein kinase	16	1
MYCN_HUMAN	N-myc proto-oncogene protein	15	1
BAZ2A_HUMAN	Bromodomain adjacent to zinc finger domain protein 2A	15	1
ZN835_HUMAN	Zinc finger protein 835	14	1

Supplementary Table 2. Correlations between USP44 expression levels and clinical features in patients with locoregionally advanced nasopharyngeal carcinoma.

Characteristic	Low Expression Group (%) *n*=235	High Expression Group (%) *n*=141	*P*-value
Age			
≤45	126 (116.9)	61 (70.1)	0.0518
>45	109 (118.1)	80 (70.9)	
Gender			
Male	177 (176.3)	105 (105.8)	0.8536
Female	58 (58.8)	36 (35.3)	
WHO type			
IIa	12 (11.9)	7 (7.1)	0.9515
IIb	223 (223.1)	134 (133.9)	
TNM Stage			
III	134 (133.1)	79 (79.9)	0.8508
IV	101 (101.9)	62 (61.1)	
VCA-IgA			
<1:80	35 (32.5)	17 (19.5)	0.4404
≥1:80	200 (202.5)	124 (121.5)	
EA-IgA			
<1:10	49 (47.5)	27 (28.5)	0.6907
≥1:10	186 (187.5)	114 (112.5)	
Locoregional recurrence			
Yes	59 (43.1)	10 (25.9)	**1.20E-05**
No	176 (191.9)	131 (115.1)	
Disease			
Yes	122 (92.5)	26 (55.5)	**1.26E-10**
No	113 (142.5)	115 (85.5)	
Death			
Yes	95 (71.3)	19 (42.8)	**3.71E-8**
No	140 (163.8)	122 (98.3)	

The *P* values were determined using the two-tailed χ^2 test. Abbreviations: WHO type IIa, differentiated non-keratinizing nasopharyngeal carcinoma; WHO type IIb, undifferentiated non-keratinizing nasopharyngeal carcinoma; VCA-IgA, viral capsid antigen immunoglobulin A; EA-IgA, early antigen immunoglobulin A.

Supplementary Table 3. List of primers used in this study.

Gene	Sequence (5' to 3')
**BSP PCR primers**	
USP44-F	GGGAATGGTTTTAGGAAGTTGA
USP44-R	CTTACCCCCCTTCTCTCCAT
**BSP sequencing primer**	
USP44	GGTTTTAGGAAGTTGATTT
**Real time RT-PCR primers**	
USP44-F	TCAGGTTACATGTCTTGCATGTG
USP44-R	TACGCTTTGAGTTACACTGGT
Ku80-F	TTGTCCAGGATGGAATTACTCTG
Ku80-R	TCAAATACAGCTGCTGTGTC
TRIM25-F	AAAGCCACCAGCTCACATCCGA
TRIM25-R	GCGGTGTTGTAGTCCAGGATGA
GAPDH-F	TGATGACATCAAGAAGGTGG
GAPDH-R	TTGTCATACCAGGAAATGAGC
**shRNA sequences**	
shRNA-*USP44*-1-F	CCGGCGGCAGGAATTGGAGTATCAACTCGAGTTGATA CTCCAATTCCTGCCGTTTTTG
shRNA-*USP44*-1-R	AATTCAAAAACGGCAGGAATTGGAGTATCAACTCGAG TTGATACTCCAATTCCTGCCG
shRNA-*USP44*-2-F	CCGGCCTCGTCTAATGAAATCCTTACTCGAGTAAGGAT TTCATTAGACGAGGTTTTTG
shRNA-*USP44*-2-R	AATTCAAAAACCTCGTCTAATGAAATCCTTACTCGAGT AAGGATTTCATTAGACGAGG
**siRNA sequences**	
siRNA-*TRIM25*	CAGCAAGCTTCCCACGTTT
siRNA-*Ku70*	GTTCTATGGTACCGAGAAA
**sgRNA sequences**	
sgRNA-*USP44*-1-F	CACCGTATCACTGCACAACTCGTAG
sgRNA-*USP44*-1-R	AAACCTACGAGTTGTGCAGTGATAC
sgRNA-*USP44*-2-F	CACCGTATTGGCCTTCGTTTAACTG
sgRNA-*USP44*-2-R	AAACCAGTTAAACGAAGGCCAATAC
sgRNA-*USP44*-3-F	CACCGTTGTTGGGCGTAACCACGAA
sgRNA-*USP44*-3-R	AAACTTCGTGGTTACGCCCAACAAC
sgRNA-*TRIM25*-1-F	CACCGAACACGGTGCTGTGCAACG
sgRNA-*TRIM25*-1-R	AAACCGTTGCACAGCACCGTGTTC
sgRNA-*TRIM25*-2-F	CACCGAGCCGGTCACCACTCCGTG
sgRNA-*TRIM25*-2-R	AAACCACGGAGTGGTGACCGGCTC
sgRNA-*TRIM25*-3-F	CACCGACCCAGCCCGAATGCCCAGG
sgRNA-*TRIM25*-3-R	AAACCCTGGGCATTCGGGCTGGGTC

Supplementary Table 4. List of antibodies used in this study.

Antibody	Company	Catalog no.	Dilution
**Western blot**			
Alpha Tubulin Antibody, Mouse Monoclonal	Proteintech	66031-1-Ig	1:1000
Anti-GAPDH antibody, Mouse Monoclonal	Sigma	G8795	1:2000
Anti-USP44 Antibody, Rabbit Polyclonal	Abcam	ab205032	1:200
XRCC5/Ku80 Antibody, Rabbit Polyclonal	Proteintech	16389-1-AP	1:500
Ku70 Antibody, Rabbit Polyclonal	Proteintech	10723-1-AP	1:500
TRIM25 Antibody, Rabbit Polyclonal	Proteintech	12573-1-AP	1:500
Anti-HA antibody produced in rabbit	Sigma	H6908	1:1000
ANTI-FLAG® M2 antibody produced in mouse	Sigma	F1804	1:500
MYC-Tag Antibody, Rabbit Polyclonal	Proteintech	16286-1-AP	1:2000
Phospho-Histone H2A.X (Ser139) (20E3), Rabbit mAb	CST	9718S	1:1000
Anti-mouse IgG, HRP-linked Antibody	CST	7076S	1:5000
Anti-rabbit IgG, HRP-linked Antibody	CST	7074S	1:5000
**Immunohistochemistry**			
TRIM25 Antibody, Rabbit Polyclonal	Proteintech	12573-1-AP	1:100
XRCC5/Ku80 Antibody, Rabbit Polyclonal	Proteintech	16389-1-AP	1:100
Caspase3 antibody, Rabbit Polyclonal	Genetex	GTX110543	1:200
Anti-USP44 Antibody, Rabbit Polyclonal	Abcam	ab205032	1:100
**Immunofluorescence**			
Anti-HA antibody produced in mouse	Sigma	H3663	1:400
XRCC5 Antibody, Mouse Monoclonal	Proteintech	66546-1-Ig	1:2000
XRCC5/Ku80 Antibody, Rabbit Polyclonal	Proteintech	16389-1-AP	1:80
TRIM25 Antibody, Rabbit Polyclonal	Proteintech	12573-1-AP	1:60
Phospho-Histone H2A.X (Ser139) (20E3), Rabbit mAb	CST	9718S	1:150
Donkey anti-mouse, Alexa Fluor ®488 IgG secondary antibody	Life	A21202	1:1000
Donkey anti-rabbit, Alexa Fluor ®594 IgG secondary antibody	Life	A21207	1:1000
**Co-Immunoprecipitation**			
Anti-HA antibody produced in rabbit	Sigma	H6908	3ug
ANTI-FLAG® M2 antibody produced in mouse	Sigma	F1804	3ug
MYC-Tag Antibody, Rabbit Polyclonal	Proteintech	16286-1-AP	3ug
Normal Mouse IgG	Invitrogen	10400C	3ug
Normal Rabbit IgG	Invitrogen	10500C	3ug
TRIM25 Antibody, Rabbit Polyclonal	Proteintech	12573-1-AP	3ug
