# Supplementary material for: USP44 regulates irradiation-induced DNA double-strand break repair and suppresses tumorigenesis in nasopharyngeal carcinoma
Source: Nat Commun. 2022 Jan 25;13:501. doi: 10.1038/s41467-022-28158-2 (PMC8789930; doi:10.1038/s41467-022-28158-2)
Supplement: Supplementary file 3 — Reporting Summary [file 41467_2022_28158_MOESM3_ESM.pdf]

nature portfolio

Corresponding author(s): Na Liu
Last updated by author(s): Dec 5, 2021

Reporting Summary

Nature Portfolio wishes to improve the reproducibility of the work that we publish. This form provides structure for consistency and transparency in reporting. For further information on Nature Portfolio policies, see our Editorial Policies and the Editorial Policy Checklist.

Statistics

For all statistical analyses, confirm that the following items are present in the figure legend, table legend, main text, or Methods section.

n/a	Confirmed	
☐	☒	The exact sample size (*n*) for each experimental group/condition, given as a discrete number and unit of measurement
☐	☒	A statement on whether measurements were taken from distinct samples or whether the same sample was measured repeatedly
☐	☒	The statistical test(s) used AND whether they are one- or two-sided<br>*Only common tests should be described solely by name; describe more complex techniques in the Methods section.*
☒	☐	A description of all covariates tested
☒	☐	A description of any assumptions or corrections, such as tests of normality and adjustment for multiple comparisons
☐	☒	A full description of the statistical parameters including central tendency (e.g. means) or other basic estimates (e.g. regression coefficient) AND variation (e.g. standard deviation) or associated estimates of uncertainty (e.g. confidence intervals)
☐	☒	For null hypothesis testing, the test statistic (e.g. *F*, *t*, *r*) with confidence intervals, effect sizes, degrees of freedom and *P* value noted<br>*Give P values as exact values whenever suitable.*
☒	☐	For Bayesian analysis, information on the choice of priors and Markov chain Monte Carlo settings
☒	☐	For hierarchical and complex designs, identification of the appropriate level for tests and full reporting of outcomes
☒	☐	Estimates of effect sizes (e.g. Cohen's *d*, Pearson's *r*), indicating how they were calculated

Our web collection on statistics for biologists contains articles on many of the points above.

Software and code

Policy information about availability of computer code

Data collection	The cell cycle distribution was detected using an ACEA NovoCyte flow cytometer and analysed with NovoExpress 1.3.0 software. The apoptosis rate was detected using a cytoFLEX flow cytometer and analysed with CytExpert 2.2 software. The quantitation of tail moments was analysed with CaspLab-Comet Assay Software.
Data analysis	All statistical analyses were performed using the SPSS version 22.0 statistical software and Graph-Pad Prism version 6.0.1 software.

For manuscripts utilizing custom algorithms or software that are central to the research but not yet described in published literature, software must be made available to editors and reviewers. We strongly encourage code deposition in a community repository (e.g. GitHub). See the Nature Portfolio guidelines for submitting code & software for further information.

Data

Policy information about availability of data

All manuscripts must include a data availability statement. This statement should provide the following information, where applicable:

- Accession codes, unique identifiers, or web links for publicly available datasets
- A description of any restrictions on data availability
- For clinical datasets or third party data, please ensure that the statement adheres to our policy

The microarray data used in this study are available in the Gene Expression Omnibus (GEO; http://www.ncbi.nlm.nih.gov/geo/) under accession codes GSE12452, GSE52068 and GSE62336. The data used in this study for gene expression profiling interactive analysis (GEPIA; http://gepia.cancer-pku.cn/index.html) are available in The Cancer Genome Atlas (TCGA; https://tcga-data.nci.nih.gov/). All the other data supporting the findings of this study are available within the article and its Supplementary Information files. The key raw data have been deposited to Research Data Deposit public platform (https://www.researchdata.org.cn/), with an approval number of RDDB2021760690. Source data are provided with this paper.

Field-specific reporting

Please select the one below that is the best fit for your research. If you are not sure, read the appropriate sections before making your selection.

- [x] Life sciences
- [ ] Behavioural & social sciences
- [ ] Ecological, evolutionary & environmental sciences

For a reference copy of the document with all sections, see nature.com/documents/nr-reporting-summary-flat.pdf

Life sciences study design

All studies must disclose on these points even when the disclosure is negative.

Sample size	Sample sizes for each experiment are provided in figure legends. For in vitro assay, n=3. For in vivo assay, usually n=6 or n=10 mice were used. No statistical method was used to determine the sample size. The sample sizes are determined empirically, and are similar in size to most existing studies in the field.
Data exclusions	No data were exluded from the analyses.
Replication	All attempts at replications were successful. Number of independent experiments and replicates are in the Statistics and Reproducibility section and the Figure legends section.
Randomization	The samples used in this study were randomly allocated into control or experimental groups.
Blinding	The investigators were not blinded to sample allocation during experiment and outcome assessment, because results used were obtained using objective quantitative methods.

Reporting for specific materials, systems and methods

We require information from authors about some types of materials, experimental systems and methods used in many studies. Here, indicate whether each material, system or method listed is relevant to your study. If you are not sure if a list item applies to your research, read the appropriate section before selecting a response.

Materials & experimental systems

n/a	Involved in the study	
☐	☒	Antibodies
☐	☒	Eukaryotic cell lines
☒	☐	Palaeontology and archaeology
☐	☒	Animals and other organisms
☐	☒	Human research participants
☒	☐	Clinical data
☒	☐	Dual use research of concern

Methods

n/a	Involved in the study	
☒	☐	ChIP-seq
☐	☒	Flow cytometry
☒	☐	MRI-based neuroimaging

Antibodies

Antibodies used

Western blot
Alpha Tubulin Antibody, Mouse Monoclonal Proteintech 66031-1-Ig 1:1000
Anti-GAPDH antibody, Mouse Monoclonal Sigma G8795 1:2000
Anti-USP44 Antibody, Rabbit Polyclonal Abcam ab205032 1:200
XRCC5/Ku80 Antibody, Rabbit Polyclonal Proteintech 16389-1-AP 1:500
Ku70 Antibody, Rabbit Polyclonal Proteintech 10723-1-AP 1:500
TRIM25 Antibody, Rabbit Polyclonal Proteintech 12573-1-AP 1:500
Anti-HA antibody produced in rabbit Sigma H6908 1:1000
ANTI-FLAG® M2 antibody produced in mouse Sigma F1804 1:500
MYC-Tag Antibody, Rabbit Polyclonal Proteintech 16286-1-AP 1:2000
Phospho-Histone H2A.X (Ser139) (20E3), Rabbit mAb CST 9718S 1:1000
Anti-mouse IgG, HRP-linked Antibody CST 7076S 1:5000
Anti-rabbit IgG, HRP-linked Antibody CST 7074S 1:5000
Immunohistochemistry
TRIM25 Antibody, Rabbit Polyclonal Proteintech 12573-1-AP 1:100
XRCC5/Ku80 Antibody, Rabbit Polyclonal Proteintech 16389-1-AP 1:100
Caspase3 antibody, Rabbit Polyclonal Genetex GTX110543 1:200
Anti-USP44 Antibody, Rabbit Polyclonal Abcam ab205032 1:100
Immunofluorescence
Anti-HA antibody produced in mouse Sigma H3663 1:400
XRCC5 Antibody, Mouse Monoclonal Proteintech 66546-1-Ig 1:2000

XRCC5/Ku80 Antibody, Rabbit Polyclonal Proteintech 16389-1-AP 1:80
TRIM25 Antibody, Rabbit Polyclonal Proteintech 12573-1-AP 1:60
Phospho-Histone H2A.X (Ser139) (20E3), Rabbit mAb CST 9718S 1:150
Donkey anti-mouse, Alexa Fluor ®488 IgG secondary antibody Life A21202 1:1000
Donkey anti-rabbit, Alexa Fluor ®594 IgG secondary antibody Life A21207 1:1000
Co-Immunoprecipitation
Anti-HA antibody produced in rabbit Sigma H6908 3ug
ANTI-FLAG® M2 antibody produced in mouse Sigma F1804 3ug
MYC-Tag Antibody, Rabbit Polyclonal Proteintech 16286-1-AP 3ug
Normal Mouse IgG Invitrogen 10400C 3ug
Normal Rabbit IgG Invitrogen 10500C 3ug
TRIM25 Antibody, Rabbit Polyclonal Proteintech 12573-1-AP 3ug

Validation

All antibodies were validated by western blot, immunohistochemistry, immunofluorescence or co-immunoprecipitation prior to isotope-polymer conjugation. Additional supporting documentation, references, and validation statements are available at the manufacturer's website.

Eukaryotic cell lines

Policy information about cell lines

Cell line source(s)

The immortalized normal human nasopharyngeal epithelial cell lines NP69 and human NPC cell lines (SUNE1, CNE1, CNE2, HNE1 and HONE1) were provided and authenticated by Professor Musheng Zeng (Sun Yat-sen University Cancer Center, China). HEK293T cell line was obtained from the American Type Tissue Culture Collection (ATCC).

Authentication

None of the cell lines were authenticated.

Mycoplasma contamination

All the cells were tested negative for mycoplasma contamination, and cultured for less than 2 months.

Commonly misidentified lines
(See ICLAC register)

None.

Animals and other organisms

Policy information about studies involving animals; ARRIVE guidelines recommended for reporting animal research

Laboratory animals

Female BALB/c nude mice (6–8 weeks old) were purchased from Charles River Laboratories (Beijing, China) and housed in barrier facilities on a 12 h light/dark cycle at temperature 18–22 °C and humidity 50–60%.

Wild animals

The study did not involve wild animals.

Field-collected samples

The study did not involve samples collected from the field.

Ethics oversight

All experimental protocols were approved by the Institutional Animal Care and Use Committee of Sun Yat-Sen University (approval number: L102012016030B) and complied with the Declaration of Helsinki.

Note that full information on the approval of the study protocol must also be provided in the manuscript.

Human research participants

Policy information about studies involving human research participants

Population characteristics

The clinical features of patients participated in the study were showed in Supplementary Table 1.

Recruitment

We randomly collected 19 fresh-frozen NPC specimens and 17 normal nasopharyngeal epithelial specimens, as well as 376 paraffin-embedded locoregionally advanced NPC specimens between January 2006 and December 2009, from Sun Yat-sen University Cancer Center (Guangzhou, China). There is no potential self-selection bias or other biases.

Ethics oversight

Our study was approved by the Institutional Ethical Review Boards of Sun Yat-sen University Cancer Center and the requirement for informed consent was waived by the ethics review boards (approval number: GZR2015-060). This study was conducted according to the REporting recommendations for tumour MARKer prognostic studies (REMARK) guidelines.

Note that full information on the approval of the study protocol must also be provided in the manuscript.

Flow Cytometry

Plots

Confirm that:

☒ The axis labels state the marker and fluorochrome used (e.g. CD4-FITC).

☒ The axis scales are clearly visible. Include numbers along axes only for bottom left plot of group (a 'group' is an analysis of identical markers).

☒ All plots are contour plots with outliers or pseudocolor plots.

☒ A numerical value for number of cells or percentage (with statistics) is provided.

Methodology

Sample preparation	The Cell Cycle and Apoptosis Kit (Keygen Biotech) was applied to detect the cell cycle distribution and apoptosis rate of each sample. For cell cycle analysis, serum-starved cells were collected 8 h after 6-Gy IR or no IR, washed in PBS and fixed in 70% ice-cold ethanol overnight. After washing, each sample was stained with 500 µl RNase A: PI (1:9, v/v) dyeing solution and screened. For apoptosis analysis, cells were collected 24 h after 6-Gy IR or no IR and washed twice with PBS. Each sample was resuspended in 500 µl binding buffer, screened and incubated with 5 µl Annexin V-FITC and 5 µl PI fluorescent dyes. FITC-/PI- cells were considered viable cells, FITC+/PI- cells were considered early apoptotic cells and FITC+/PI+ cells were considered late apoptotic or dead cells. For the NHEJ reporter assay, the EJ5-GFP plasmid was generously provided by Professor Muyan Cai (Sun Yat-sen University Cancer Center, China). The indicated cells were seeded in 6-well plates, transfected with EJ5-GFP and infected with I-SceI-expressing adenovirus after 18 h. The medium was replaced after 14 h to avoid adenovirus toxicity. Cells were harvested after 72 h.
Instrument	The cell cycle distribution was detected using an ACEA NovoCyte flow cytometer. The apoptosis rate and GFP-positive cells were detected using a cytoFLEX flow cytometer.
Software	The cell cycle distribution was analysed with NovoExpress 1.3.0 software. The apoptosis rate and GFP-positive cells were analysed with CytExpert 2.2 software.
Cell population abundance	10000 cells in P2 gates were counted for cell cycle analysis. 20,000 cells were counted for apoptosis analysis. 10,000 cells were counted for NHEJ reporter assay.
Gating strategy	For cell cycle analysis, the percentage of PE-A-positive cells was determined upon gating strategy: i) FSC-A vs SSC-A to isolate cells from debris, ii) PE-A vs PE-H to isolate single cells and iii) PE-A for detection of PE-A positive population. For apoptosis analysis, the percentage of viable cells, early apoptotic cells or late apoptotic cells were determined upon gating strategy: i) FSC-A vs SSC-A to isolate cells from debris, ii) FSC-A vs FSC-H to isolate single cells and iii) FITC-A vs PI-A to detect the population of viable cells, early apoptotic cells or late apoptotic cells. For GFP-positive cells analysis, the percentage of GFP-positive cells was determined upon gating strategy: i) FSC-A vs SSC-A to isolate cells from debris, ii) FSC-Width vs FSC-A to isolate single cells and iii) FSC-A vs FITC-A for detection of GFP-positive population.

☒ Tick this box to confirm that a figure exemplifying the gating strategy is provided in the Supplementary Information.
